# Rethinking the Use of Routine Platelet Transfusions for Head Injured Patients on Antiplatelet Therapy

**DOI:** 10.7759/cureus.6136

**Published:** 2019-11-12

**Authors:** Gabriel O Ologun, Arpitha Pamula, Mojirola Alegbejo-Olarinoye, Paul Granet, Robert Behm

**Affiliations:** 1 General Surgery, Robert Packer Hospital/Guthrie Clinic, Sayre, USA; 2 Medicine, St. Bonaventure University, Robert Packer Hospital/Guthrie Clinic, Sayre, USA; 3 General Surgery, University-College of Health Sciences, Abuja, NGA; 4 Trauma/Surgical Critical Care, Robert Packer Hospital/Guthrie Clinic, Sayre, USA; 5 Trauma/Critical Care, Robert Packer Hospital/Guthrie Clinic, Sayre, USA

**Keywords:** head injury, antiplatelet therapy, head trauma, transfusion

## Abstract

Traumatic brain injury is responsible for over one million hospital visits, and thousands of deaths annually. The aging population is associated with an increased use of anticoagulation and antiplatelet agents which complicates traumatic brain injury. The use of antiplatelet agents significantly increases baseline risk of intracranial hemorrhage. However, routine platelet transfusion in an attempt to reverse the effects of antiplatelet agents may be detrimental. Here, we report a case of an elderly woman with mild traumatic brain injury, who suffered a tragic demise after platelet transfusion.

## Introduction

Traumatic brain injury (TBI) is responsible for over one million hospital visits, 200,000 admissions and 50,000 deaths annually. The aging population is associated with an increased use of anticoagulation and antiplatelet agents, which complicates TBI. The combination of age over 65 years and the use of antiplatelet agents significantly increases the baseline risk of intracranial hemorrhage [1]. Both anticoagulation and antiplatelet agents have been shown to have increased bleeding and increased mortality in patients with intracranial hemorrhage [2,3]. We report a case of an elderly patient with mild TBI, who suffered a tragic demise after platelet transfusion.

## Case presentation

The patient was a 99-year-old woman, who suffered a mechanical fall from standing height with no reported loss of consciousness. She was initially transported by the emergency medical service to an outside hospital emergency department, where airway, breathing and circulation were noted to be without any abnormality. Vitals signs were normal and she was on room air. She had no focal neurologic deficits, and her Glasgow Coma Scale score (GCS) was 15.

Her past medical history included dementia, hypertension, coronary artery disease, gastroesophageal reflux disease, diabetes mellitus, and atrial fibrillation. She had a past surgical history that included cholecystectomy, inguinal herniorrhaphy, and total abdominal hysterectomy. She had no known allergies to medication, and her only home medication was baby aspirin 81 mg daily. She was a nonsmoker and did not consume alcohol. She lived in an assisted living facility and ambulated with a rolling walker.

**Figure 1:**
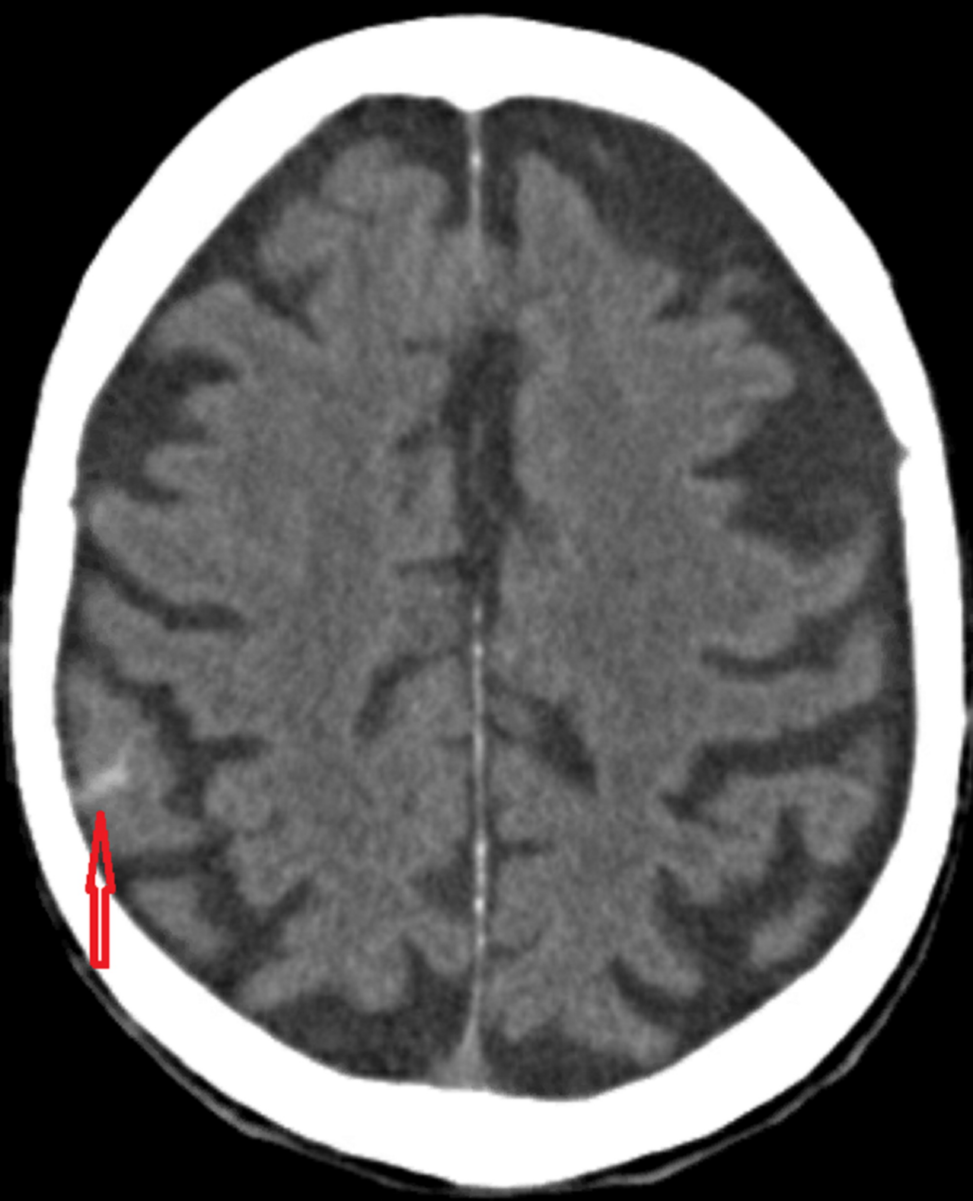
Noncontrast computed tomography scan of the head, axial view showing small focus of subarachnoid hemorrhage (arrow). No evidence of midline shift.

**Figure 2:**
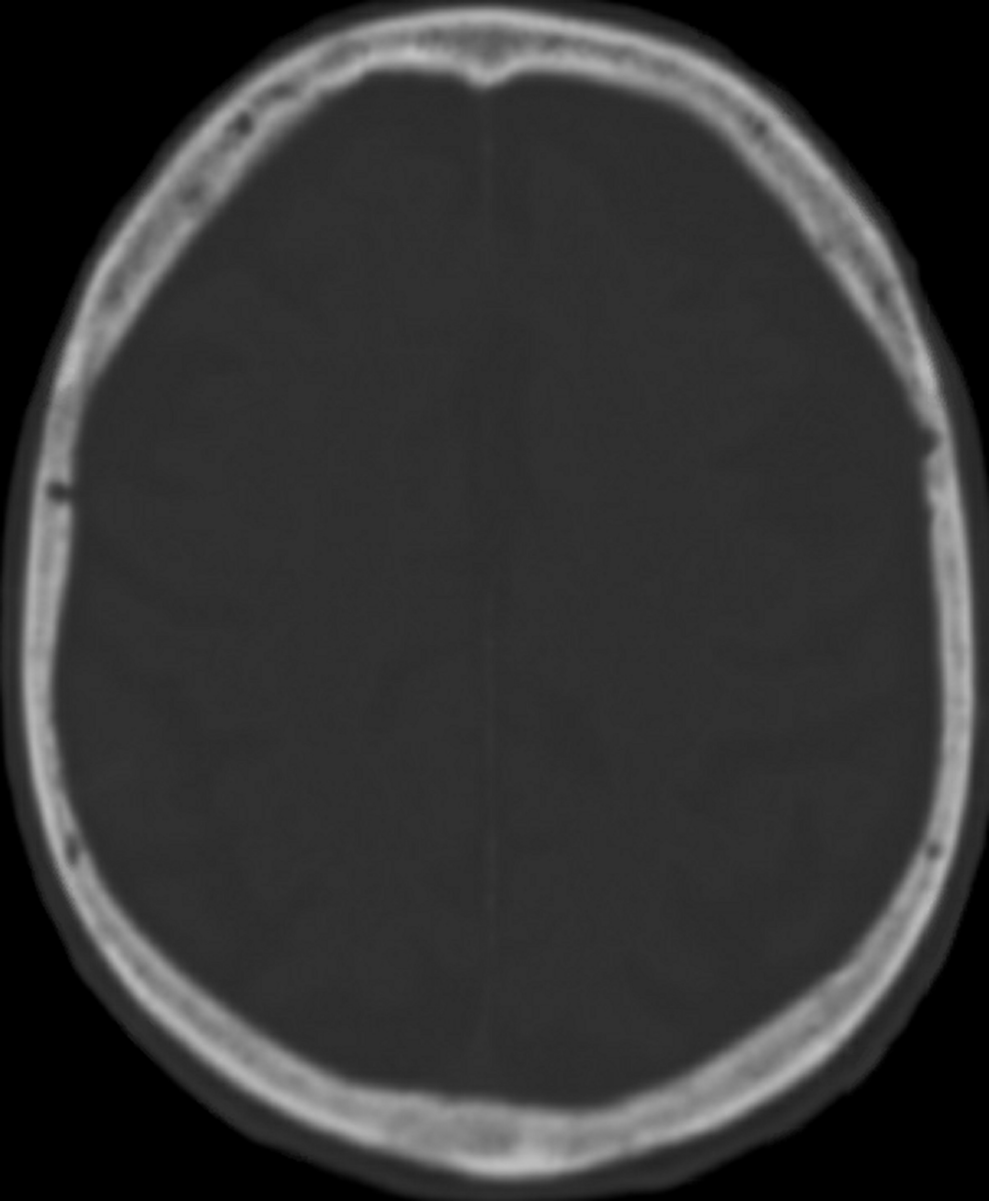
Axial view of a computed tomography scan of the calvaria, bone window, no evidence of depressed skull fracture.

Computed tomography scan (CT) of the head and neck was performed which showed a tiny subarachnoid hemorrhage (SAH) on the right parietal-occipital area (Figure 1). There was no evidence of calvarial fracture (Figure 2). There was also no evidence of cervical spine fracture. The patient was subsequently transferred to our level II trauma center for further workup and management.

On arrival to our emergency department trauma bay approximately four hours after the initial trauma, she had no change in her neurologic status. Her vital signs were pulse 74 beats per minute, blood pressure 118 mmHg systolic, respiratory rate 18 breaths per minute, temperature 98.2 degrees Fahrenheit, and saturation of 96% on two liters of nasal cannula. Her abdomen was soft, nontender, nondistended, and without ecchymosis. Focused assessment sonography for trauma (FAST) was performed, which was negative. Pelvic x-ray and chest x-ray were both unremarkable. CT scan images from the outside hospital were reviewed.

Laboratory investigation was significant for mild anemia and hyperglycemia, otherwise unremarkable (Table 1).

**Table 1:** Laboratory values

Complete Blood Count (CBC)	Data	Reference Range and Units
White blood cell (WBC) count	7.8	4.0-11.0 K/uL
Hemoglobin (Hgb)	12.6	14.0-18.0 g/dL
Hematocrit (Hct)	36.1	40.0-54.0%
Platelet count	179	140-440 K/uL

Basic Metabolic Profile (BMP)	Data	Reference Range and Units
Sodium	140	136-145 mEq/L
Potassium	3.9	3.5-5.1 mEq/L
Chloride	109	98-107 mEq/L
Carbon dioxide	18	22-29 mEq/L
Blood urea nitrogen (BUN)	23	6-23 mg/dL
Creatinine	0.94	0.67-1.17 mg/dL
Glucose	148	70-99 mg/dL

Based on the hospital protocol for a SAH patient on an antiplatelet agent, she was transfused a unit of platelets. She was also administered desmopressin. Shortly thereafter, she became hypotensive, bradycardic, and hypoxic, with saturations in the 50s. Atropine was administered for bradycardia, and she was intubated for airway protection. Resuscitative efforts using crystalloid and vasopressor drugs were initiated, while repeat FAST and chest x-ray were negative. She rapidly declined hemodynamically, ultimately progressing to asystole. Advanced cardiac life support was initiated, and after three cycles of cardiopulmonary resuscitation, the patient was declared dead. Autopsy was not performed.

## Discussion

The widespread use of antiplatelet agents in the TBI population has resulted in the empiric use of platelet transfusion. However, this practice is controversial given the known side effects of platelet transfusion, including blood groups type (ABO) incompatibility reactions, sepsis, arrhythmia, transfusion-related acute lung injury, stroke, and death [2]. We recently encountered a 99-year-old woman, who took a daily baby aspirin, and suffered a very mild TBI. Her GCS was 15, and her contusion was tiny. Despite her clinical presentation, and a four-hour interval between injury and presentation, she did not get repeat imaging. She was transfused platelets because it was the hospital’s policy. Approximately 40 minutes following initiating platelet transfusion, and 10 minutes following transfusion completion, she rapidly deteriorated hemodynamically, and died 30 minutes later. Platelet transfusion was the only significant intervention this patient was given, so it was the most likely explanation for demise.

The indications, risks, and benefits of platelet transfusion should be carefully deliberated, prior to administration, in patients who are taking antiplatelet medication, and suffered a TBI. Below is a review of the impact platelet transfusion has on platelet function, size of intracranial hemorrhage, mortality, and long-term outcome.

Various assays have been used to determine the impact of aspirin on platelet function, and they clearly show a decrease in platelet aggregation when introduced to aspirin. Using the Aspirin Response Test (ART) which is 100% sensitive and 96% specific for determining aspirin-related platelet inhibition, it has been shown that 64% of patients given platelet transfusions for a TBI had reversal of inhibition based on the ART; however, the outcome of those patients was not discussed [4]. Another study, although not focused on a trauma patient population, found that patients who transfused platelets within 12 hours of symptoms of spontaneous bleed had similar platelet activity to a patient not exposed to aspirin [2]. Despite this physiologic response to platelet transfusion, the authors noted adverse effects of the platelet transfusion and suggested that these risks were not worth the potential benefit of restoring normal platelet aggregation.

Although it appears reversal of platelet inhibition can be achieved with platelet transfusion, it is the clinical response to the transfusion that should impact our decision making. When overall mortality was investigated in 328 patients on antiplatelet therapy with TBI, the mortality of the patients transfused with platelets was not significantly different from those patients not transfused (17.5% vs 16.7%, P=0.85) [5]. Others have shown an increased mortality in those patients with intracranial hemorrhage on antiplatelet agents who were transfused platelets. These patients were comparatively older with a lower GCS but the mortality difference was significant (30% vs 16%, P=0.01). There also appeared to be a higher rate of platelet transfusions as the severity of injury increased [6]. The clinical impact of platelet transfusion on patients with mild TBI (GCS≥13) and intracranial hemorrhage who were on antiplatelet agents has also be investigated looking at rates of neurologic decline, medical decline, injury progression on imaging, and overall mortality. A total of 108 patients were included in this study with 41% receiving platelets and 59% not being transfused. There was no statistically significant difference in the rate of neurologic decline, need for surgical intervention, and progression of injury based on imaging, cardiac events, respiratory events, or GCS. There was a trend, though not significant, towards an increased rate of medical decline, in patients who received platelet transfusion [7]. A similar study looked at TBI in 109 elderly patients (over age 50 years) who were taking antiplatelet agents; however, their inclusion criteria comprised more severe head injuries to include a GCS as low as 7. They stratified head injury into grades I-IV using CT scan, and found that pre-injury antiplatelet use was associated with a 20% rate of grade III-IV injury, compared to historic rates of 7% in patients not taking antiplatelet agents. There was a 4.5% rate of hemorrhage progression in grade I and II injury with a mortality of 1%. The mortality of the patients without progression of bleed was 7% suggesting mortality was secondary to other injuries. Approximately 73% of those with grade III-IV injuries were taken urgently to the operating room, while the remaining 27% were admitted for comfort care. Mortality associated with head injury in patients taking warfarin was correlated with progression of hemorrhage with reversal of international normalization ratio reducing hemorrhage progression and improving mortality. Conversely, the severity of the initial injury seems to be related to mortality in those patients taking antiplatelet agents rather than progression of hemorrhage [8].

Holzmacher et al. conducted a multi-institutional observational study in six United States trauma centers where they investigated the efficacy of platelet transfusion to reverse antiplatelet medication and to evaluate how it improves outcome in patients with TBI. Of the 66 patients enrolled, 89% were on aspirin. They found that platelet transfusion significantly decreased the platelet inhibition due to aspirin (76±30.2% to 52±32.5%, P<0.01), but not associated with change in outcomes in patient on antiplatelet medication following TBI. Platelet transfusion was associated with longer length of stay (7.8 vs 3.5 days, P<0.01), but there were no differences in mortality [9].

The acuity of trauma and the life-saving maneuvers often performed can distract from the reality that long-term disability is the most common consequence of injury, which is also true for TBI. Long-term follow-up in patients with TBI using the Glasgow Outcome Score Extended (GOSE) and the Functional Status Examination (FSE) has shown that patients who received platelet transfusions did not have significantly different GOSE or FSE scores when compared to patients who did not receive platelet transfusions [10].

Transfusing platelets to patients taking antiplatelet agents who have suffered a TBI has been shown to be ineffective at improving clinical outcomes. Despite this being a seemingly intuitive therapy with basic science research suggesting transfusion can restore platelet activity to normal, this could be a dangerous practice. This case was meticulously reviewed and part of the review process involved an investigation into the individual who donated the platelets. This person was a male with a long history of platelet donation (over 50 units) and no history of allergic reactions following transfusion. Prior to her transfusion, she was appropriately cross matched. The unit of platelets was also cultured to make sure her decline was not a result of gram-negative bacteremia. The culture results were negative. The only intervention given to this woman prior to her sudden death was the platelet transfusion, and her death was associated with the platelet transfusion.

## Conclusions

Platelet transfusion should be carefully considered prior to administration in patients taking antiplatelet agents who suffered a TBI, as its use is not without adverse effects. In addition, no clear clinical benefits have been seen when platelets are transfused to this patient population. Hence, very careful consideration needs to be exercised prior to implementing this intervention. In our case, the patient suffered a tragic demise after platelet transfusion. In our practice, we no longer routinely transfuse platelets to patients on antiplatelet therapy who sustained a TBI with intracranial hemorrhage.
